# Supplementary material for: Imaging the master regulator of the antioxidant response in non-small cell lung cancer with positron emission tomography
Source: bioRxiv. 2023 Dec 17:2023.12.16.572007. Preprint. [Version 1] doi: 10.1101/2023.12.16.572007 (PMC10760199; doi:10.1101/2023.12.16.572007)
Supplement: Supplement 1 [file media-1.pdf]

EXTENDED FIGURES AND LEGENDS

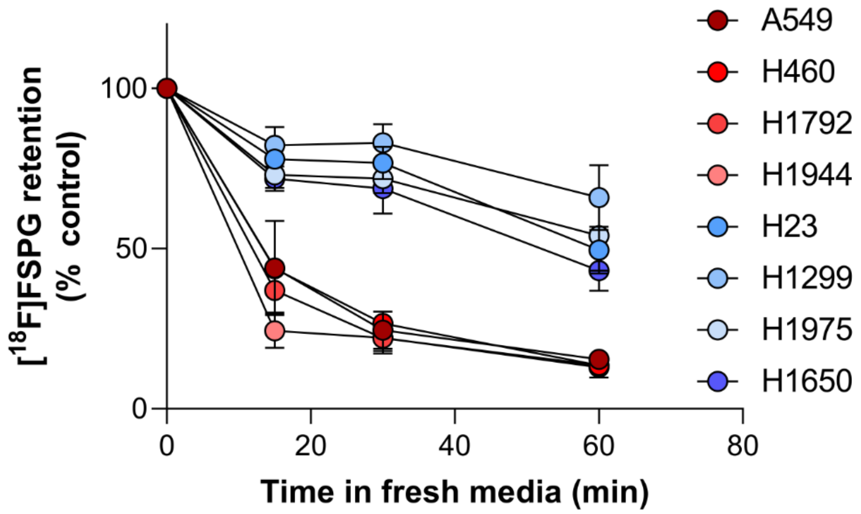

Extended Figure 1. Time course of [^{18}F]FSPG efflux across NRF2-high (red) and NRF2-low NSCLC cells (blue).

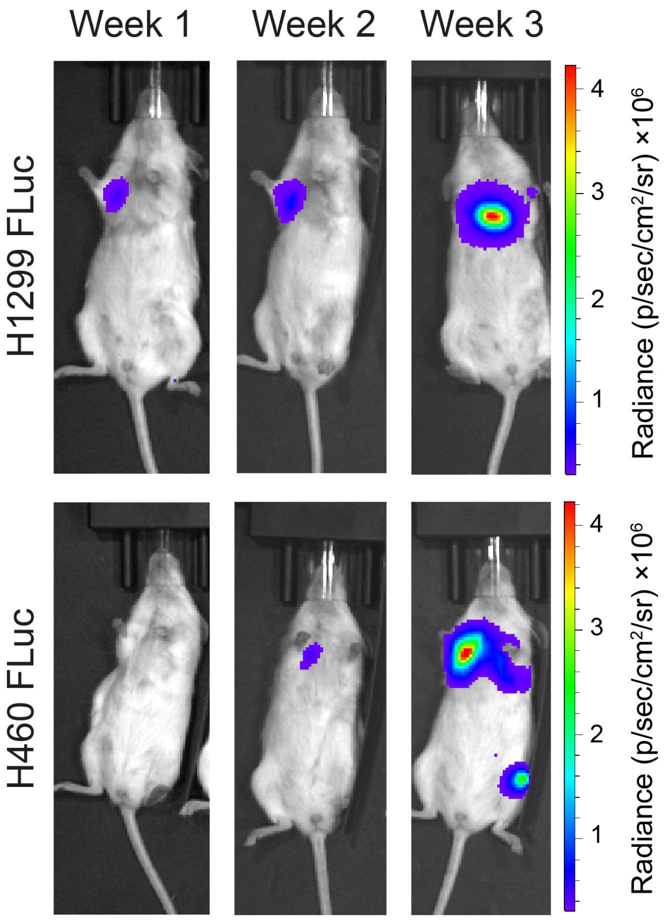

Extended Figure 2. Representative BLI from mice orthotopically implanted with H460 FLuc and H1299 FLuc tumour cells.

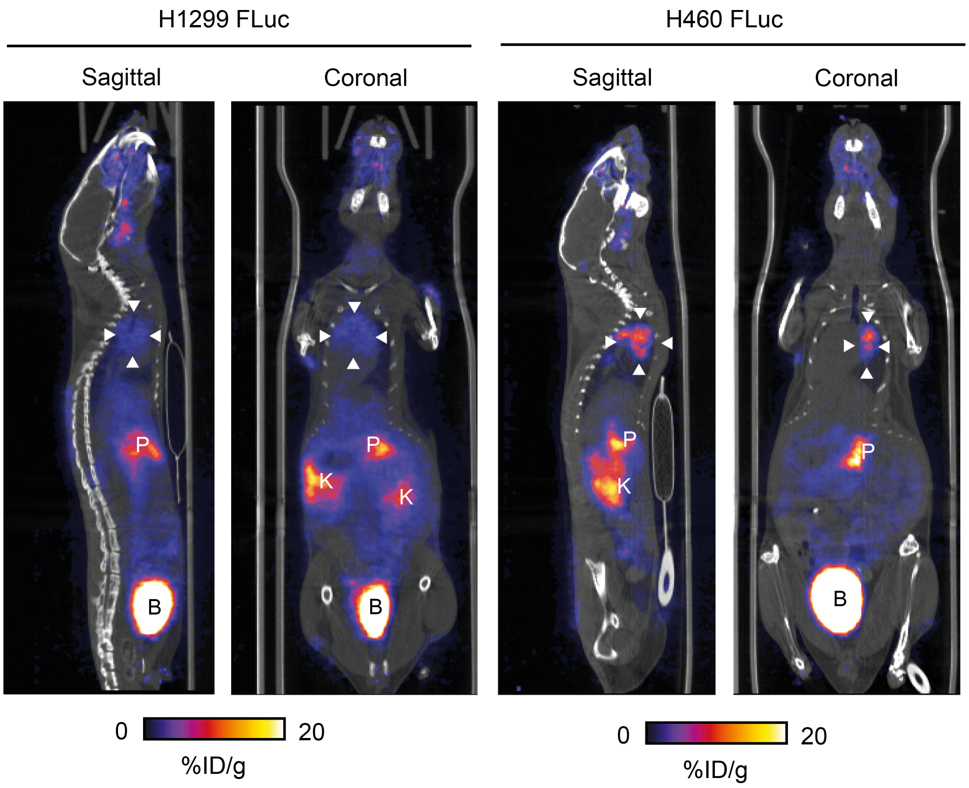

Extended Figure 3. Representative single slice PET/CT images from mice orthotopically implanted with H460 FLuc and H1299 FLuc tumour cells. Arrowheads denote the tumour. K, kidney; P, pancreas; B, bladder.

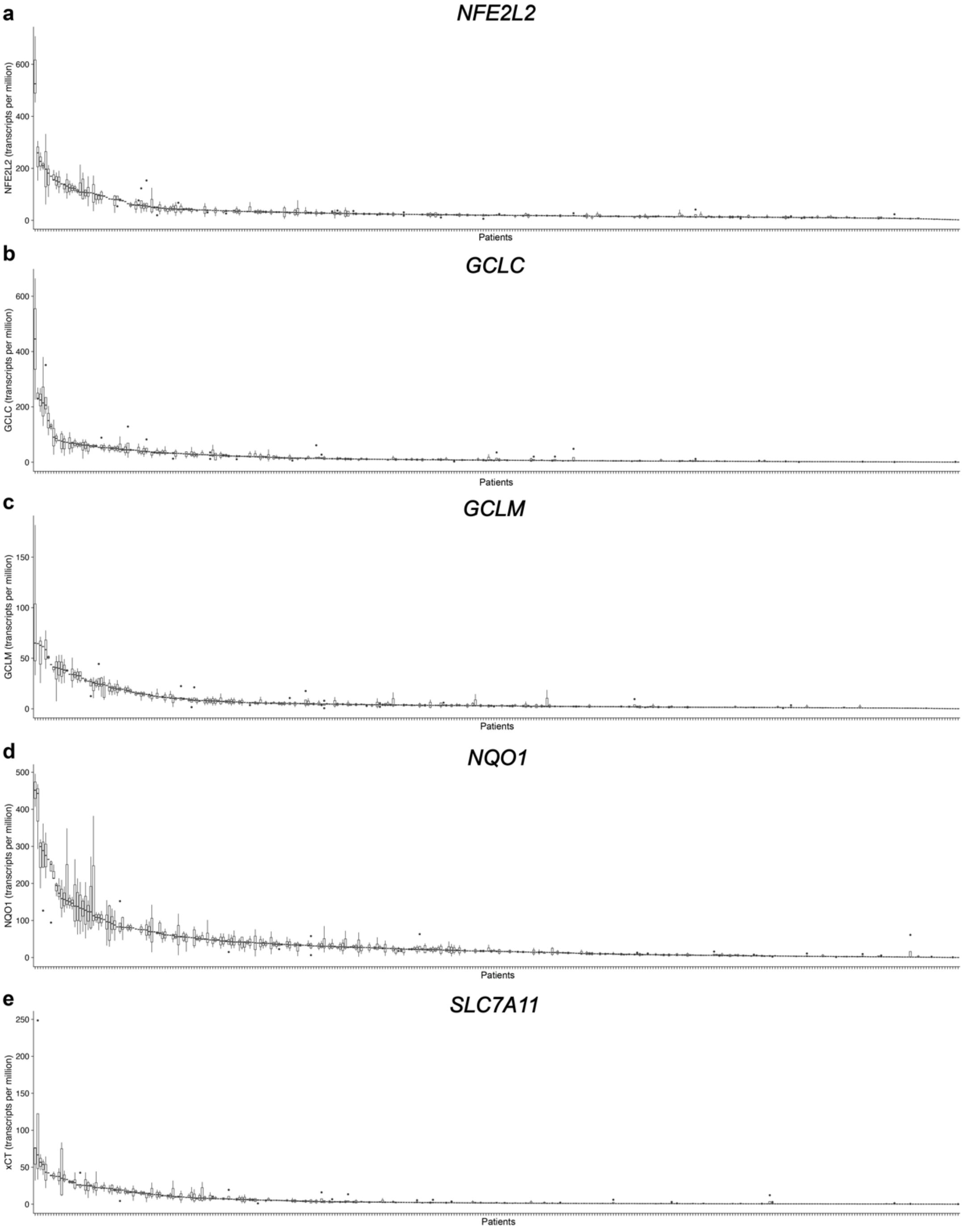

Extended Figure 4. Multi-region bulk RNA sequencing data from 882 samples from 344 NSCLC patients. Plots show expression (transcripts per million) of *NFE2L2* (a), *GCLC* (b), *GCLM* (c), *NQO1* (d) and *SLC7A11* (e). Box plots show the lower and upper quartiles (box limits) and the median (centre line). The length of whiskers is 1.5 × Interquartile range and outlier points outside this range are shown. Patients are ordered from highest to lowest median expression of each gene along the x-axis, and thus are not in the same order between plots.

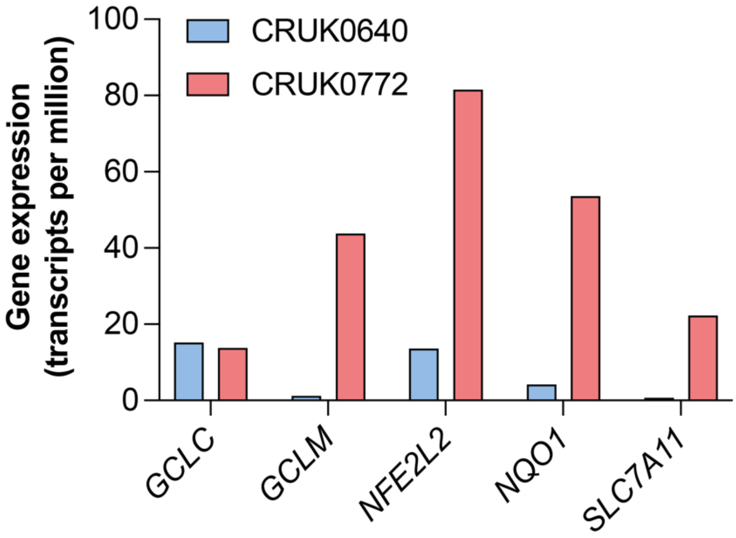

Extended Figure 5. Mean NRF2-regulated gene expression in CRUK0640 and CRUK0772 tumour resection samples from TRACERx421 RNA sequencing data.

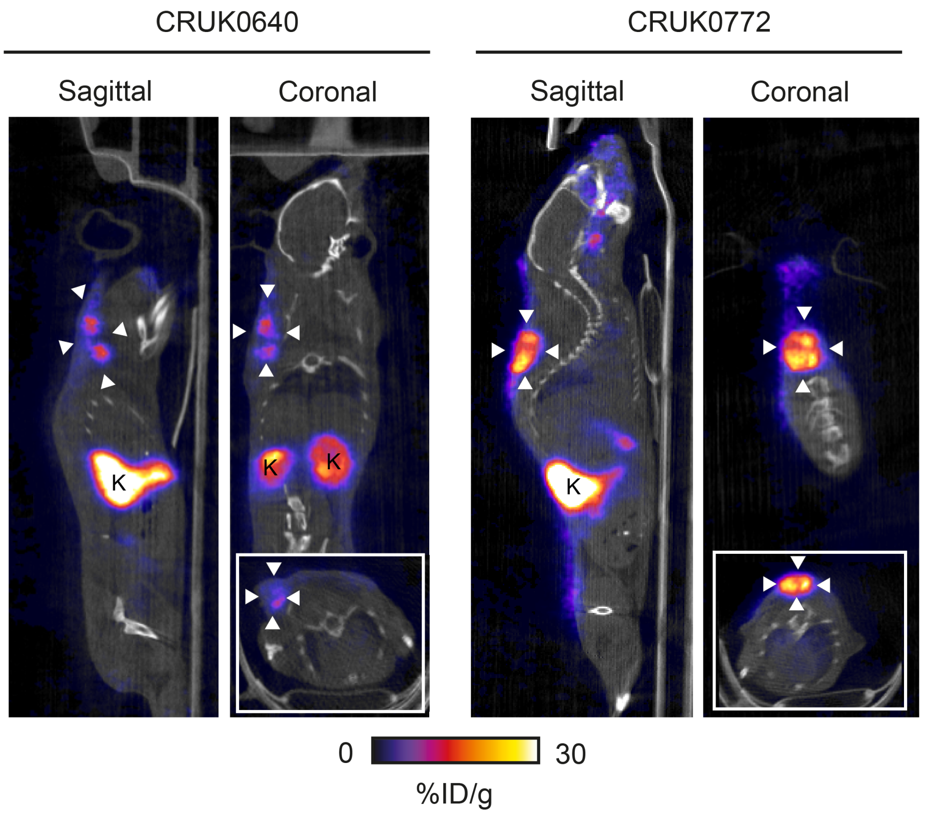

Extended Figure 6. Representative single slice PET/CT images from mice bearing CRUK0640 or CRUK0772 patient-derived xenografts. Arrowheads denote the tumour. K, kidney.

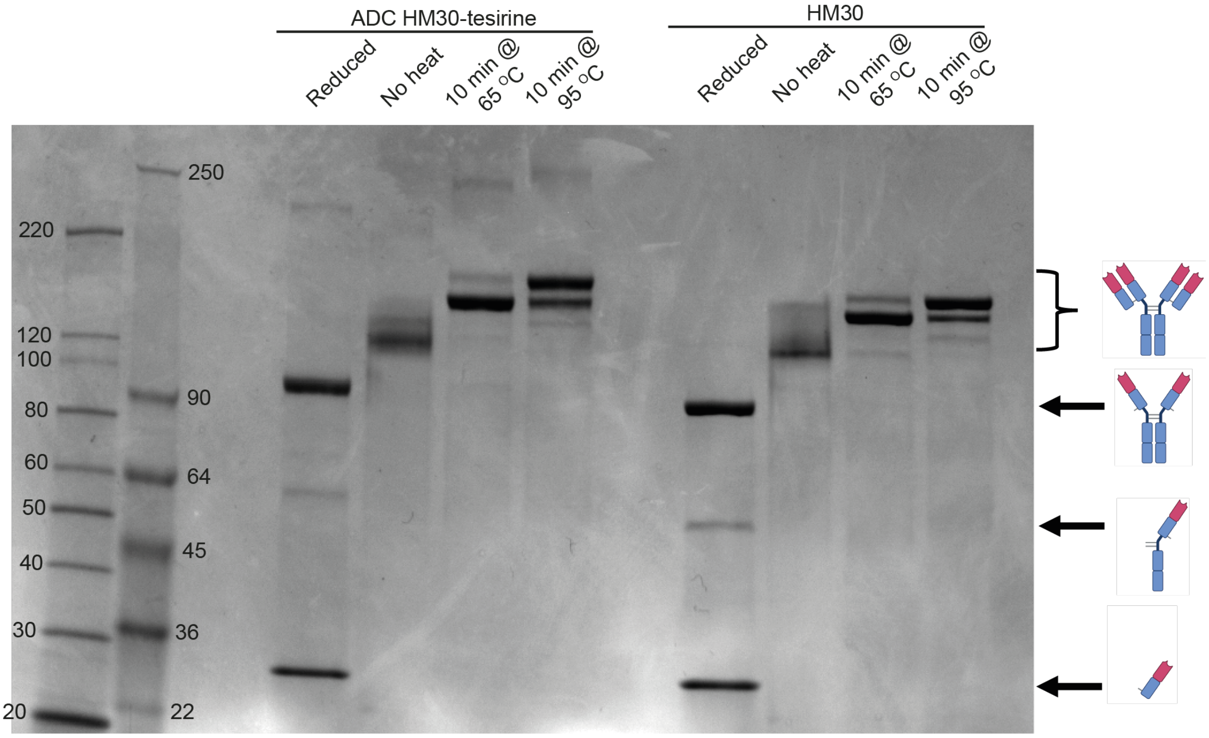

Extended Figure 7. HM30-tesirine and unconjugated HM30 monoclonal antibody SDS-PAGE profile with a range of pre-treatment conditions.

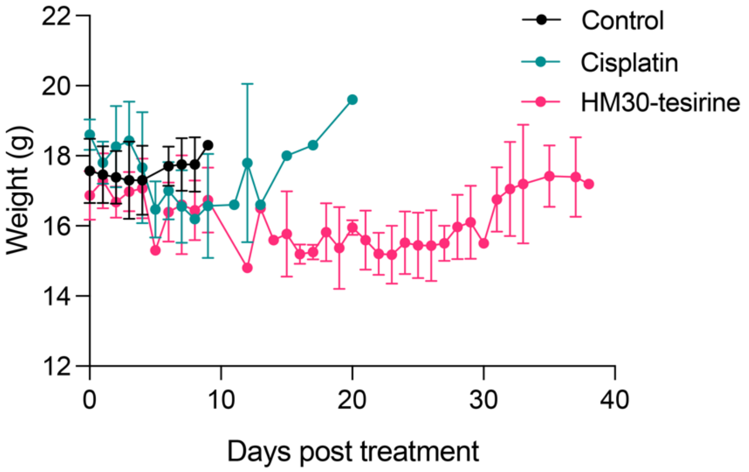

Extended Figure 8. Body weight from H460 tumour-bearing mice following treatment with vehicle control, cisplatin, and HM30-tesirine.
